# Supplementary material for: Effects of Tai Chi on Postural Control in People with Peripheral Neuropathy: A Systematic Review with Meta-Analysis
Source: Healthcare (Basel). 2023 May 26;11(11):1559. doi: 10.3390/healthcare11111559 (PMC10252344; doi:10.3390/healthcare11111559)
Supplement: Supplementary file 1 [file healthcare-11-01559-s001.zip › Literature retrieval strategy.docx]

**Table S1. Search strategy for China National Knowledge Infrastructure (CNKI), China VIP Full-text Database (VIP), Google Scholar, Web of Science databases, Science Direct (SD), Wanfang Data Knowledge Service Platform, and Springer Link**

| **Search** | **Query** | **China National Knowledge Infrastructure (CNKI)** | | **China VIP Full-text Database (VIP)** | | **Google Scholar** | | **Web of Science** | | **Science Direct (SD)** | | **Wanfang Data Knowledge Service Platform** | | **Springer Link** | | **Others** |
| --- | --- | --- | --- | --- | --- | --- | --- | --- | --- | --- | --- | --- | --- | --- | --- | --- |
| **retrieval time** |  | ***1** | ***2** | ***1** | ***2** | ***1** | ***2** | ***1** | ***2** | ***1** | ***2** | ***1** | ***2** | ***1** | ***2** |  |
| #1 | TI=(Tai Chi or Tai chi training or taijiquan or t'ai chi ch'uan) OR TI=(Polyneuropathy or disorders of peripheral nerves or PN) OR TI=(postural control or equilibrium or pose control or balance) | 657 | 32 | 674 | 225 | 489 | 88 | NA | NA | 234 | 41 | 1193 | 93 | NA | NA |  |
| #2 | AB=(Tai Chi or Tai chi training or taijiquan or t'ai chi ch'uan) OR AB=( Polyneuropathy or disorders of peripheral nerves or PN) OR AB=(postural control or equilibrium or pose control or balance) | 568 | 156 | 767 | 232 | 568 | 109 | NA | NA | 342 | 19 | 968 | 166 | NA | NA |  |
| #3 | S1 OR S2 | 548 | 92 | 312 | 34 | 399 | 88 | NA | NA | 321 | 24 | 565 | 112 | NA | NA |  |
| #4 | S1 AND S2 | 467 | 59 | 304 | 20 | 387 | 20 | NA | NA | 245 | 35 | 456 | 156 | NA | NA |  |
| #5 | S3 AND S4 | 467 | 81 | 311 | 35 | 299 | 23 | NA | NA | 220 | 2 | 298 | 14 | NA | NA |  |
| #6 | (Tai Chi OR Tai chi training OR taijiquan OR t'ai chi ch'uan) ANG (PN OR Polyneuropathy OR disorders of peripheral nerves) ANG (postural control OR equilibrium OR pose control OR balance ) | NA | NA | NA | NA | NA | NA | 20,468 | 0 | NA | NA | NA | NA | 5463 | 879 |  |
| #7 | (MM "disorders of peripheral nerves") OR (MM "Polyneuropathy") OR (MM "PN") | NA | NA | NA | NA | NA | NA | 2546 | 66 | NA | NA | NA | NA | 3121 | 300 |  |
| #8 | S6 AND S7 | NA | NA | NA | NA | NA | NA | 312 | 324 | NA | NA | NA | NA | 203 | 213 |  |
| Filters | English and Chinese Language | 467 | 81 | 336 | 10 | 299 | 23 | 312 | 12 | 220 | 2 | 298 | 14 | 202 | 11 |  |
| Filters | Human | 359 | | | | | | | | | | | | | | |
| **ClinialTrials.Gov** |  |  | |  | |  | |  | |  | |  |  | |  | |
| Condition: | Peripheral neuropathy in diabetes |  | |  | |  | |  | |  | |  |  | |  | |
| Other Terms: | Cross Education |  | |  | |  | |  | |  | |  | 359 | |  | |
| Study Type: | Intervention |  | |  | |  | |  | |  | |  | 359 | |  | |
| Status: | Completed |  | |  | |  | |  | |  | |  | **10** | |  | |
| **Other** |  |  | |  | |  | |  | |  | |  |  | |  | |
|  | Additional articles identified, checking reference lists |  | |  | |  | |  | |  | |  |  | | **2** | |

Note: The retrieval time ”*1” is on February 22, 2022; time” *2” is from February 22, 2022, to April 28, 2023.
