# Supplementary material for: Effects of Tai Chi on Postural Control in People with Peripheral Neuropathy: A Systematic Review with Meta-Analysis
Source: Healthcare (Basel). 2023 May 26;11(11):1559. doi: 10.3390/healthcare11111559 (PMC10252344; doi:10.3390/healthcare11111559)
Supplement: Supplementary file 1 [file healthcare-11-01559-s001.zip › Literature search equation.doc]

Note: Each database has two searches.

==============================

Database:

China National Knowledge Infrastructure (CNKI):Result numbers and search dates:

(467 results on February 22 ,2022; 548 results on April 28, 2023;)

Search:

#1TI=(Tai Chi or Tai chi training or taijiquan or t'ai chi ch'uan) OR TI=(peripheral neuropathy or Polyneuropathy or disorders of peripheral nerves or PN) OR TI=(postural control or equilibrium or pose control or balance)

#2AB=(Tai Chi or Tai chi training or taijiquan or t'ai chi ch'uan) OR AB=(peripheral neuropathy or Polyneuropathy or disorders of peripheral nerves or PN) OR AB=(postural control or equilibrium or pose control or balance)

#3(#1 OR #2)

#4 #1 AND #2

#5 (#1 OR #2) AND #4

==============================

Database:

China VIP Full-text Database (VIP): Result numbers and search dates:

(336 results on February 22, 2022;346 results on April 28, 2023)

Search:

#1TI=(Tai Chi or Tai chi training or taijiquan or t'ai chi ch'uan) OR TI=(peripheral neuropathy or Polyneuropathy or disorders of peripheral nerves or PN) OR TI=(postural control or equilibrium or pose control or balance)

#2AB=(Tai Chi or Tai chi training or taijiquan or t'ai chi ch'uan) OR AB=(peripheral neuropathy or Polyneuropathy or disorders of peripheral nerves or PN) OR AB=(postural control or equilibrium or pose control or balance)

#3(#1 OR #2)

#4 #1 AND #2

#5 (#1 OR #2) AND #4

==============================

Database:

Google Scholar: Result numbers and search dates:

(299 results on February 22, 2022;322 results on April 28, 2023)

Search:

#1TI=(Tai Chi or Tai chi training or taijiquan or t'ai chi ch'uan) OR TI=(peripheral neuropathy or Polyneuropathy or disorders of peripheral nerves or PN) OR TI=(postural control or equilibrium or pose control or balance)

#2AB=(Tai Chi or Tai chi training or taijiquan or t'ai chi ch'uan) OR AB=(peripheral neuropathy or Polyneuropathy or disorders of peripheral nerves or PN) OR AB=(postural control or equilibrium or pose control or balance)

#3(#1 OR #2)

#4 #1 AND #2

#5 (#1 OR #2) AND #4

==============================

Database:

Web of Science databases: Result numbers and search dates:

(312 results on February 22, 2022; 324 results on April 28, 2023)

Search:

#1 (Tai Chi OR Tai chi training OR taijiquan OR t'ai chi ch'uan) ANG (PN OR peripheral neuropathy in diabetes OR Polyneuropathy OR disorders of peripheral nerves) ANG (postural control OR equilibrium OR pose control OR balance )

#2 (MM "disorders of peripheral nerves") OR (MM "Polyneuropathy") OR (MM "PN") OR (MM "peripheral neuropathy in diabetes ")

#3 #1 AND #2

==============================

Database:

Wanfang Data Knowledge Service Platform: Result numbers and search dates:

(298 results on February 22, 2022; 312 results on April 28, 2023)

Search:

#1TI=(Tai Chi or Tai chi training or taijiquan or t'ai chi ch'uan) OR TI=(peripheral neuropathy in diabetes or Polyneuropathy or disorders of peripheral nerves or PN) OR TI=(postural control or equilibrium or pose control or balance)

#2AB=(Tai Chi or Tai chi training or taijiquan or t'ai chi ch'uan) OR AB=(peripheral neuropathy in diabetes or Polyneuropathy or disorders of peripheral nerves or PN) OR AB=(postural control or equilibrium or pose control or balance)

#3(#1 OR #2)

#4 #1 AND #2

#5 (#1 OR #2) AND #4

==============================

Database:

Science Direct (SD): Result numbers and search dates:

(220 results on February 22, 2022;222 results on April 28, 2023)

Search:

#1TI=(Tai Chi or Tai chi training or taijiquan or t'ai chi ch'uan) OR TI=(peripheral neuropathy or Polyneuropathy or disorders of peripheral nerves or PN) OR TI=(postural control or equilibrium or pose control or balance)

#2AB=(Tai Chi or Tai chi training or taijiquan or t'ai chi ch'uan) OR AB=(peripheral neuropathy or Polyneuropathy or disorders of peripheral nerves or PN) OR AB=(postural control or equilibrium or pose control or balance)

#3(#1 OR #2)

#4 #1 AND #2

#5 (#1 OR #2) AND #4

==============================

Database:

Springer Link: Result numbers and search dates:

(202 results on February 22, 2022; 213 results on April 28, 2023)

#1 (Tai Chi OR Tai chi training OR taijiquan OR t'ai chi ch'uan) ANG (PN OR peripheral neuropathy in diabetes OR Polyneuropathy OR disorders of peripheral nerves) ANG (postural control OR equilibrium OR pose control OR balance )

#2 (MM "disorders of peripheral nerves") OR (MM "Polyneuropathy") OR (MM "PN") OR (MM "peripheral neuropathy in diabetes ")

#3 #1 AND #2
